# Utilization of Mao (*Antidesma thwaitesianum* Muell. Arg.) Pomace Meal to Substitute Rice Bran on Feed Utilization and Rumen Fermentation in Tropical Beef Cattle

**DOI:** 10.3390/vetsci9110585

**Published:** 2022-10-23

**Authors:** Nirawan Gunun, Pichad Khejornsart, Sineenart Polyorach, Chatchai Kaewpila, Thachawech Kimprasit, Ittipol Sanjun, Anusorn Cherdthong, Metha Wanapat, Pongsatorn Gunun

**Affiliations:** 1 Department of Animal Science, Faculty of Technology, Udon Thani Rajabhat University, Udon Thani 41000, Thailand; 2 Faculty of Natural Resources and Agro-Industry, Kasetsart University Chalermphrakiat Sakon Nakhon Province Campus, Sakon Nakhon 47000, Thailand; 3 Department of Animal Production Technology and Fisheries, Faculty of Agricultural Technology, King Mongkut’s Institute of Technology Ladkrabang, Bangkok 10520, Thailand; 4 Department of Animal Science, Faculty of Natural Resources, Rajamangala University of Technology Isan, Sakon Nakhon Campus, Sakon Nakhon 47160, Thailand; 5 Tropical Feed Resources Research and Development Center (TROFREC), Department of Animal Science, Faculty of Agriculture, Khon Kaen University, Khon Kaen 40002, Thailand

**Keywords:** energy source, digestibility, rumen fermentation, mao pomace meal, beef cattle

## Abstract

**Simple Summary:**

Mao by-products have the potential to be used as ruminant energy feedstuffs to reduce feed costs. We assessed the effect of replacing rice bran in concentrate diets with mao pomace meal on the feed utilization and rumen fermentation characteristics in beef cattle. The present findings found that replacement of rice bran with mao pomace meal improves the rumen fermentation characteristics without any negative effects on feed intake, whereas it decreases nutrient digestibility. Therefore, mao pomace meal could be used as an energy feedstuff in the diet of tropical beef cattle.

**Abstract:**

This experiment was conducted to investigate the effects of replacing rice bran with mao pomace meal on feed intake, digestibility, and rumen fermentation in beef cattle. Four crossbred (50% Brahman × 50% Thai native) beef cattle with an initial body weight of 195 ± 13 kg and 16 months of age were used in a 4 × 4 Latin square design. The dietary treatments included four levels of RB replacement with mao pomace meal at 0, 33, 67, and 100% in concentrate diets. Rice straw was used as a roughage source, fed ad libitum. Replacement of mao pomace meal with rice bran did not affect (*p* > 0.05) the intakes of concentrate, rice straw, and total dry matter intake. Ether extract intake decreased linearly when increasing the levels of mao pomace meal (*p* < 0.01). The experimental diets had no effect (*p* > 0.05) on the digestibility of fiber and crude protein, while dry matter, organic matter, and ether extract digestibility decreased linearly in the group of mao pomace meal replacing rice bran (*p* < 0.05). Increasing levels of mao pomace meal in concentrate diets did not alter rumen pH, ammonia–nitrogen, or total volatile fatty acid concentration (*p* > 0.05). The proportion of propionate increased linearly (*p* < 0.05), whereas acetate and the acetate to propionate ratio decreased linearly (*p* < 0.05) when replacing rice bran with mao pomace meal. Moreover, the proportion of propionate was greatest, while acetate was lowest when mao pomace meal was included at 100% in the concentrate diet. In conclusion, the replacement of rice bran with mao pomace meal in a diet could enhance the efficiency of rumen fermentation. Nonetheless, it reduced the digestion of nutrients in tropical beef cattle.

## 1. Introduction

The use of agro-industrial by-products in ruminant feeding is on the rise globally because it reduces feed costs, manipulates rumen microbiota, fermentation, and animal products’ carbon footprint [1,2]. Rice bran (RB) is a by-product produced in the rice milling industry [3]. The RB is the most widely used energy source in animal diets and it is also a source of oil for human consumption [4,5]. However, the price of RB (0.35 USD) has been high, affecting the cost of livestock feed in Southeast Asia, especially Thailand. Feeding costs can be reduced by substituting RB with other low-cost energy feed additives.

*Antidesma thwaitesianum* Muell. Arg. (Thai local name: Mao or Mamao) is classified in the family Phyllanthaceae and distributed in Australia, Southeast Asia, and Africa [6,7]. Mao pomace consists of the seeds and skins that remain after mao has been produced for juice and wine in the food industry; and it is seen as an environmental problem [8,9]. The mao pomace consists of 8.6–11.6% crude protein (CP), 2.6–3.8% ether extract (EE), 9.2% condensed tannins (CT) [8,10], and 3865 kcal/kg gross energy (GE) [11]. Hence, mao pomace could be a good source of energy and plant secondary compounds in animal feed. In previous studies, it was found that the supplementation of mao pomace meal (MPM) at 200 g/head/day resulted in improved ruminal fermentation and reduced methane production without adverse effects on feed intake, digestibility, hematology, milk yield, and milk composition in dairy cows [8]. In an in vitro study, Gunun et al. (unpublished) found that MP supplementation improved ruminal fermentation by reducing protozoal population, methane production, and ammonia–nitrogen (NH_3_-N) concentration. In addition, Gunun et al. [12] reported that the addition of mao seed meal enhanced nitrogen utilization and ruminal fermentation, especially propionate, and had no effect on nutrient digestibility. However, the use of MPM as an energy source in concentrate diets has not been investigated. Therefore, the objective of this study was to evaluate the effect of MPM in replacing RB on feed intake, digestibility, and rumen fermentation in tropical beef cattle.

## 2. Materials and Methods

### 2.1. Ethical Procedure

All the animal care and experimental procedures were approved by the Animals Ethical Committee of the Rajamangala University of Technology Isan (approval no. 20/2564).

### 2.2. Animals, Treatments, and Experimental Design

The research was conducted on the beef cattle farm of the of the Faculty of Natural Resources, Rajamangala University of Technology Isan, Sakon Nakhon Campus, Phangkhon, Sakon Nakhon, Thailand. Fresh mao pomace were provided by Sakon Nakhon Winery Ltd., Phuphan, Sakon Nakhon, Thailand. They were ground to create MPM after being sundried for approximately 3 days.

Four male crossbred (50% Brahman × 50% Thai native) beef cattle at 16 months of age and 195 ± 13 kg of initial body weight (BW). Each cattle is in a separate pen with clean water and mineral blocks available. The mineral blocks (each kg) contained NaCl, 995.11 g; Na, 390.00 g; Mg, 2.00 g; Zn, 0.81 g; Cu, 0.22 g; I, 0.10 g; and Se, 0.01 g (KNZ, Arnhem, Netherlands). The design of this study was based on a 4 × 4 Latin square design with four periods and four treatments. Each period that comprised the experiment lasted for 21 days, with 14 days assigned to treatment adaptation and feed intake assessments, and the remaining 7 days to the collection of samples. Each period was separated by a 7-day transition period. Four dietary treatments were to replace RB with MPM in the concentrate mixture at 0, 33, 67, and 100% on a dry matter (DM) basis. The cattle were fed concentrate (Table 1) at 0.5% BW, and rice straw was fed ad libitum into two equal meals at 08:00 h and 16:00 h.

### 2.3. Data Collection and Sampling Procedures

At the beginning and end of each period, the BW of every animal was measured. Every morning, feed samples of roughage and concentrate were measured by weighing the feed that was given and the feed that was uneaten. On the last five days of each period, a digestibility test was conducted with individual pens for the cattle. Fresh fecal samples (about 500 g) were obtained via rectal sampling. The samples of each animal’s daily fresh feces were pooled and then frozen. The samples (feed, refusals, and feces) were dried at 60 °C, ground (1 mm screen using Cyclotech Mill; Tecator, Hoganas, Sweden). The amount of DM, ash, EE, and CP [13]; neutral detergent fiber (NDF); acid detergent fiber (ADF) [13,14]; and acid-insoluble ash (AIA) were measured. AIA was designed to estimate the digestibility of the nutrients [15]. The vanillin–HCl method, as modified by Burns [16], was used to analyze CT. The levels of non-fibrous carbohydrates (NFC) were calculated using the equations NFC = 100 − (%CP + %NDF + %EE + %ash). [17].

A stomach tube connected to a vacuum pump was used to collect 200 mL of rumen fluid at 0 and 4 h post feeding on the final day of each period. To avoid saliva contact, the first 100 mL of the ruminal samples were thrown away. The samples were then filtered through four layers of cheesecloth, and the pH was measured quickly using a portable pH meter (FiveGo; Mettler-Toledo GmbH, Greifensee, Switzerland). The ruminal fluid samples were centrifuged at 16,000× *g* for 15 min at 4 °C, and the supernatant was stored in the freezer at –20 °C. The thawed ruminal samples were then used to test for NH_3_-N (Kjeltech Auto 1030 Analyzer, Tecator, Hoganiis, Sweden) [18] and volatile fatty acid (VFA) using high-pressure liquid chromatography [19].

### 2.4. Statistical Analysis

The general linear model (GLM) in SAS software was used to examine the variances of the data using a 4 × 4 Latin square design [20]. The model Yijk = μ + Mi + Aj + Pk + εijk was used to evaluate the data, where Yijk is the observation from treatment i, cattle j, and period k; μ is the overall mean; Mi is the mean effect of the treatments (i = 1–4); Aj is the mean effect of the cattle (j = 1–4); Pk is the mean effect of the periods (k = 1–4); and εijk is the residual error. Using orthogonal polynomial contrasts, trends in treatments were statistically compared (linear, quadratic, and cubic). A significance level of *p* < 0.05 was applied to determine whether an effect was substantial.

## 3. Results

### 3.1. Chemical Composition of the Diets

The concentrate diet was formulated by using available local feed resources and contained CP at 13.2–13.5%. The EE and NFC content was decreased, while NDF and ADF contents were increased according to the increasing MPM in concentrate (Table 2). The MPM is composed of 11.2% of CP, 1.4% of EE, 53.3% of NDF, 41.9% of ADF, 28.4% of NFC, and 9.4% of CT.

### 3.2. Feed Intake and Nutrient Digestibility

Increasing the level of MPM in concentrate as the replacement of RB did not change feed intake and nutrient intake (*p* > 0.05), except for the intake of EE (*p* < 0.01) (Table 3). The digestibility of DM, OM, and EE decreased linearly (*p* < 0.05) in the group of MPM replacing RB at 100% (Table 4).

### 3.3. Ruminal Fermentation Characteristics

Ruminal pH, NH_3_-N, total VFA, and butyrate (C4) at 0 and 4 h post feeding were similar among treatments (*p* > 0.05) (Table 5). Replacing RB with MPM at 100% was shown to linearly decrease acetate (C2), while propionate (C3) was increased linearly. Therefore, the C2:C3 ratio decreased linearly at 0 h post feeding (*p* < 0.05).

## 4. Discussion

### 4.1. The Chemical Composition of Diets

The CP content of the MPM was 11.2% (Table 2). Lokaewmanee [10] reported similar results, determining that MPM contained 11.6% CP, while Gunun et al. [8] found that MPM contained 8.6% CP. Variable CP content in MPM may be related to mao fruit and wine processing, planting area, or fertilizer. Furthermore, the CT content of MPM was found to be 9.4% in the current study, which is consistent with the findings of Gunun et al. [8], who found CT to be 9.2% in DM. Increasing levels of MPM increased NDF and ADF content in the concentrate. The MPM contained NDF and ADF at 53.3% and 41.9%, respectively. Its results showed higher fiber content in concentrate when MPM was added. However, the EE content decreased in the group of MPM replacing RB due to the MPM lower EE content (1.4% DM) in the current study, while RB had higher EE content (18.9% DM) [21].

### 4.2. Feed Intake and Nutrient Digestibility

The inclusion of plants containing tannins into animal diets usually reduces voluntary feed intake and the digestibility of nutrients. Beauchemin et al. [22] suggested that voluntary feed intake was decreased by high CT intake (>50 g/kg DM), while low concentrations usually have no effect. The lower intake of high tannin concentration results in a reduction in feed palatability [23]. Palatability is often based on the astringency associated with CT–protein complexes formed from proteins in ruminant saliva [24]. In the present study, the highest level of CT intake was at 20 g/kg DM, which is lower than the level mentioned. Hence, the inclusion of MPM in concentrate did not affect feed intake. Similarly, Gunun et al. [8] reported that MPM supplementation at 100–300 g/hd/day (or CT intake at 0.7–2.0 g/kg DM) did not affect the feed intake in dairy cows. However, the EE intake and digestibility were decreased with the inclusion of MPM to replace RB. The lower EE content of MPM in the diet had an adverse effect on the EE intake and digestibility.

The digestibility of DM and OM was decreased by the inclusion of MPM in the diets. This is plausible because tannins have a negative effect on digestibility due to the formation of tannin–carbohydrate or tannin–protein complex binding [25]. Moreover, tannins are also toxic to rumen microorganisms, particularly fibrolytic bacteria; thus, reducing fiber digestion [26]. The toxic effect is strongly dependent on the dose, molecular weight, and type of tannin [27]. However, the inclusion of MPM to replace RB did not affect the digestibility of NDF and ADF in the current study. This is in agreement with Nunoi et al. [28], who reported that replacing RB with tamarind seed meal containing CT (9.7% DM) did not affect the fiber digestibility in dairy steers.

### 4.3. Rumen Fermentation Characteristics

In the current study, the ruminal pH decreased from 6.9 to 6.7 at 4 h post feeding. The reduction in ruminal fluid pH was found when cattle were fed concentrate and roughage after feeding. Increasing MPM levels did not adversely affect the ruminal pH. Similarly, Gunun et al. [8] reported that the ruminal pH was not affected by the supplementation of MPM at 300 g/hd/d in dairy cows. In addition, the ruminal pH was in the optimum range (pH 6.5–7.0), indicating that it was an optimal level for the microbial digestion of fiber [29,30].

The concentration of NH_3_-N in the rumen increased after feeding [31,32]. The vast majority of dietary crude protein is microbially degraded to NH_3_-N in the rumen, which serves as the major nitrogen source for rumen microorganisms [33]. The CT in plants can bind to proteins to form CT–protein complexes, which decrease protein degradation and also NH_3_-N production [34,35]. Gunun et al. [8] found that supplementing MPM at 300 g/hd/d for 4 h after feeding reduced the ruminal NH_3_-N concentrations in dairy cows. In contrast, the ruminal NH_3_-N concentration was unaffected by MPM levels at 0 and 4 h post feeding in the present study, indicating that MPM had no effect on ruminal protein degradation. These results agree with Gunun et al. [12], who evaluated the supplementation of mao seed meal at 0.8–2.4% of the total DM intake in goats and also, did not observe differences in the NH_3_-N concentration. This result differs from our previous studies because the effects of CT in mao by-product on NH_3_-N concentrations differ with CT concentrations, inclusion levels, digestion kinetics of the basal diet, and ruminant species. The ruminal NH_3_-N concentrations in the current study are within the range of those found in other studies with ruminants fed mao by-product [8,12].

For ruminants, VFA has been the main energy source, providing up to 75% of the total metabolizable energy [36]. Higher NFC levels in the diets provided readily fermentable carbohydrates and higher volatile fatty acids in the rumen [37]. In the present study, the inclusion of MPM in the diets had no effect on NFC intake, and also unchanged the total VFA production in the rumen. In addition, our previous studies found that adding MPM increased propionate while decreasing acetate in lactating dairy cows [8]. Replacing RB with MPM at 100% in beef cattle improved propionate while lowering acetate and C2:C3 in the current study. Similarly, Nunoi et al. [28] found that the replacement of tammarind seed meal for RB at 100% enhanced propionate and butyrate at 4 h after feeding in dairy steers. The increase in propionate proportion was caused by the inactivity of acetogenic bacteria as a result of CT in the plant, while H_2_ utilization switched to propionate production, where free H_2_ is more favorable for propionic bacteria activity [38,39,40]. Consequently, a reduced C2:C3 ratio was found in the current study. Propionate is the main precursor of gluconeogenesis in ruminants; hence, making a substantial net contribution to their glucose production [41,42]. These data demonstrate that adding MPM to beef cattle is shown to improve VFA production in the rumen, especially propionate.

## 5. Conclusions

Replacement of RB by MPM as an energy source in concentrate diets at 100% improved the rumen fermentation characteristics and this had no effect on feed intake, whereas the digestibility of DM and OM decreased in beef cattle. Further research is required to evaluate the effect of the replacement of RB by MPM on growth performance and meat quality in tropical beef cattle.

## Figures and Tables

**Table 1:** Ingredients of the diets used in the experiment.

	Mao Pomace Meal Replacing Rice Bran, % Dry Matter			
Item	0	33	67	100
Ingredient, % dry matter				
Cassava chip	60.5	60.5	60.5	60.5
Rice bran	21.0	14.0	7.0	0.0
Mao pomace	0.0	7.0	14.0	21.0
Soybean meal	12.9	12.9	12.9	12.9
Molasses	2.0	2.0	2.0	2.0
Urea	1.6	1.6	1.6	1.6
Mineral and vitamin mixture	1.0	1.0	1.0	1.0
Salt	0.5	0.5	0.5	0.5
Sulfur	0.5	0.5	0.5	0.5

**Table 2:** Chemical composition of concentrate, rice straw, and MPM.

	Mao Pomace Meal Replacing Rice Bran, % Dry Matter				Rice Straw	Mao Pomace Meal
Item	0	33	67	100		
Chemical composition						
Dry matter, %	90.9	90.7	89.3	90.7	93.3	92.5
Organic matter, % dry matter	94.0	94.3	94.6	95.0	87.5	94.3
Crude protein, % dry matter	13.3	13.3	13.2	13.5	2.0	11.2
Ether extract, % dry matter	2.5	2.0	1.5	1.0	0.5	1.4
Neutral detergent fiber, % dry matter	30.2	32.4	34.4	35.4	70.0	53.3
Acid detergent fiber, % dry matter	18.0	19.1	20.8	21.9	56.1	41.9
Non-fiber carbohydrates, % dry matter	48.0	46.6	45.5	45.1	15.0	28.4
Ash, % dry matter	6.0	5.7	5.4	5.0	12.5	5.7
Condensed tannins, % dry matter	0.0	0.7	1.3	2.0	-	9.4
Price (USD/kg)	0.31	0.29	0.27	0.25	-	-

**Table 3:** Effect of mao pomace meal (MPM) as a substitute for rice bran (RB) in concentrate diets on feed intake in beef cattle.

	Mao Pomace Meal Replacing Rice Bran, % Dry Matter					Contrast		
Item	0	33	67	100	SEM	Linear	Quadratic	Cubic
Dry matter intake								
Rice straw								
kg/d	3.0	2.8	2.6	2.5	0.21	0.36	0.93	0.93
%BW	1.5	1.3	1.3	1.3	0.08	0.09	0.54	0.77
g/kg BW^0.75^	55.7	50.5	48.7	46.8	4.12	0.14	0.68	0.85
Concentrate								
kg/d	1.0	1.0	1.0	1.0	0.05	0.80	0.94	0.78
%BW	0.5	0.5	0.5	0.5	0.01	0.55	0.14	0.16
g/kg BW^0.75^	19.0	17.9	18.5	18.3	0.53	0.51	0.38	0.25
Total intake								
kg/d	4.0	3.7	3.6	3.4	0.25	0.42	0.93	0.90
%BW	2.0	1.8	1.8	1.7	0.12	0.06	0.45	0.63
g/kg BW^0.75^	74.7	68.4	67.2	65.1	4.64	0.13	0.62	0.75
Nutrient intake, kg/d								
Organic matter	3.6	3.4	3.3	3.1	0.22	0.43	0.95	0.91
Crude protein	0.2	0.2	0.2	0.2	0.01	0.67	0.72	0.75
Ether extract	0.04	0.03	0.03	0.02	0.001	<0.01	0.42	0.17
Neutral detergent fiber	2.4	2.3	2.2	2.1	0.18	0.42	0.93	0.94
Acid detergent fiber	1.9	1.7	1.7	1.6	0.15	0.37	0.91	0.88
Non-fiber carbohydrates	0.9	0.9	0.9	0.8	0.05	0.49	0.90	0.95

**Table 4:** Effect of mao pomace meal (MPM) as a substitute for rice bran (RB) in concentrate diets on nutrient digestibility in beef cattle.

	Mao Pomace Meal Replacing Rice Bran, % Dry Matter					Contrast		
Item	0	33	67	100	SEM	Linear	Quadratic	Cubic
Digestibility coefficients, %								
Dry matter	64.9	63.3	61.5	60.2	0.56	0.01	0.90	0.90
Organic matter	66.9	65.6	63.3	62.0	0.56	<0.01	0.88	0.82
Crude protein	60.6	58.0	56.8	54.1	2.31	0.81	0.42	0.62
Ether extract	76.0	72.2	59.6	56.5	3.29	0.04	0.79	0.42
Neutral detergent fiber	59.2	58.7	55.4	53.8	3.14	0.06	0.79	0.62
Acid detergent fiber	57.7	56.6	53.0	53.1	2.12	0.14	0.82	0.60

**Table 5:** Effect of mao pomace meal (MPM) as a substitute for rice bran (RB) in concentrate diets on rumen fermentation characteristics in beef cattle.

	Mao Pomace Meal Replacing Rice Bran, % Dry Matter					Contrast		
Item	0	33	67	100	SEM	Linear	Quadratic	Cubic
pH								
0 h post feeding	6.9	6.8	6.9	7.0	0.40	0.27	0.15	0.45
4 h post feeding	6.8	6.7	6.6	6.5	0.15	0.06	0.93	0.92
Ammonia–nitrogen (mg/dL)								
0 h post feeding	19.3	19.8	18.7	18.7	0.34	0.36	0.67	0.36
4 h post feeding	20.1	21.0	20.1	19.8	0.62	0.77	0.65	0.65
Total volatile fatty acid (mM)								
0 h post feeding	48.7	47.2	41.3	45.3	1.72	0.32	0.44	0.38
4 h post feeding	49.9	53.3	53.1	51.2	0.81	0.60	0.14	0.80
Volatile fatty acid (mol/100 mol)								
Acetate								
0 h post feeding	67.9	67.5	66.6	61.4	0.96	0.04	0.26	0.68
4 h post feeding	59.8	61.0	62.3	58.2	1.32	0.55	0.08	0.39
Propionate								
0 h post feeding	20.9	21.1	21.4	26.8	1.08	0.02	0.29	0.64
4 h post feeding	28.8	27.2	25.3	28.3	1.43	0.62	0.17	0.48
Butyrate								
0 h post feeding	11.2	11.4	11.7	11.8	0.19	0.23	0.88	0.81
4 h post feeding	11.4	11.8	11.7	13.5	0.42	0.13	0.41	0.53
Acetate to propionate ratio								
0 h post feeding	3.4	3.3	3.1	2.4	0.17	0.02	0.47	0.69
4 h post feeding	2.2	2.3	2.4	2.1	0.07	0.89	0.12	0.51

## Data Availability

Not applicable.
